# γ-Oryzanol Enhances Adipocyte Differentiation and Glucose Uptake

**DOI:** 10.3390/nu7064851

**Published:** 2015-06-15

**Authors:** Chang Hwa Jung, Da-Hye Lee, Jiyun Ahn, Hyunjung Lee, Won Hee Choi, Young Jin Jang, Tae-Youl Ha

**Affiliations:** 1 Department of Food Biotechnology, Korea University of Science and Technology, 1201 Anyangpangyo-ro, Bundang-ku, Seongnam 463-746, Korea; E-Mails: chjung@kfri.re.kr (C.H.J.); dlekgp26@nate.com (D.-H.L.); jyan@kfri.re.kr (J.A.); kfri2@daum.net (W.H.C.); 2 Metabolic Mechanism Research Group, Korea Food Research Institute, 1201 Anyangpangyo-ro, Bundang-ku, Seongnam 463-746, Korea; E-Mails: Lee.Hyun-jung@kfri.re.kr (H.L.); jyj616@kfri.re.kr (Y.J.J.)

**Keywords:** γ-oryzanol, 3T3-L1, adipocyte, glucose uptake, PPAR-γ, mTORC1

## Abstract

Recent studies show that brown rice improves glucose intolerance and potentially the risk of diabetes, although the underlying molecular mechanisms remain unclear. One of the phytochemicals found in high concentration in brown rice is γ-oryzanol (Orz), a group of ferulic acid esters of phytosterols and triterpene alcohols. Here, we found that Orz stimulated differentiation of 3T3-L1 preadipocytes and increased the protein expression of adipogenic marker genes such as peroxisome proliferator-activated receptor gamma (PPAR-γ) and CCAAT/enhanced binding protein alpha (C/EBPα). Moreover, Orz significantly increased the glucose uptake in insulin-resistant cells and translocation of glucose transporter type 4 (GLUT4) from the cytosol to the cell surface. To investigate the mechanism by which Orz stimulated cell differentiation, we examined its effects on cellular signaling of the mammalian target of rapamycin complex 1 (mTORC1), a central mediator of cellular growth and proliferation. The Orz treatment increased mTORC1 kinase activity based on phosphorylation of 70-kDa ribosomal S6 kinase 1 (S6K1). The effect of Orz on adipocyte differentiation was dependent on mTORC1 activity because rapamycin blocks cell differentiation in Orz-treated cells. Collectively, our results indicate that Orz stimulates adipocyte differentiation, enhances glucose uptake, and may be associated with cellular signaling mediated by PPAR-γ and mTORC1.

## 1. Introduction

Insulin is an important anabolic hormone that promotes cell differentiation of 3T3-L1 preadipocytes by activating adipocyte-specific transcription factors including peroxisome proliferator-activated receptor-γ (PPAR-γ) and CCAAT/enhancer-binding protein α (C/EBPα) [1]. PPAR-γ has emerged as a potent insulin sensitizer and is used in the treatment of type 2 diabetes [2,3]. PPAR-γ is dramatically induced during adipogenesis and expressed predominantly in adipose tissue [4]. It is critical for the functions of mature adipocytes, including in lipid metabolism, adipokine secretion, and insulin sensitivity [5]. High-affinity ligands of PPAR-γ induce insulin-sensitizing factors such as adiponectin [6] and fibroblast growth factor 21 (FGF-21) [7]. Rosiglitazone is one of the most potent PPAR-γ agonists in adipose tissues, which has prolonged anti-diabetic effects but causes serious side effects. In contrast, natural products, which have proven to be a promising resource for drug discovery, are believed to have minimal side effects.

The compound γ-oryzanol (Orz), an important bioactive component present mainly in the bran layers and germ of rice [8], is composed of more than 10 similar ferulate esters [9]. Orz has been investigated for various biological activities including anti-oxidative, anti-inflammatory, and anti-cancer [10,11]. Recent finding show that brown rice may improve hyperglycemia compared with white rice in diabetic complications [12,13,14,15], indicating that Orz, a component of brown rice, may reduce blood glucose concentrations in type 2 diabetes. Kozuka and colleagues reported that Orz ameliorates ER stress-induced β-cell dysfunction and apoptosis [16]. A recent paper showed that Orz directly enhances glucose-stimulated insulin secretion in pancreatic islets [17]. This evidence suggests that the consumption of brown rice and Orz may improve some aspects of glycemic control in diabetes. However, its exact molecular mechanism is not fully understood.

In this study, we investigated whether Orz regulates cell differentiation of 3T3-L1 preadipocytes via upregulation of adipogenic markers including PPAR-γ. We also investigated the effects of Orz on glucose uptake and translocation of glucose transporter type 4 (GLUT4).

## 2. Materials and Methods

### 2.1. Materials

Dulbecco’s modified Eagle’s medium (DMEM), fetal bovine serum (FBS), bovine calf serum (CS) and penicillin-streptomycin were obtained from Gibco BRL (Grand Island, NY, USA). Anti-β-actin (sc-47778) and secondary antibodies were purchased from Santa Cruz Biotechnology (Santa Cruz, CA, USA). Antibodies against p-mTOR (#5536s), mTOR (#9272), p-S6K1 (#9205), S6K1 (#9202), PPAR-γ (#2435), and C/EBP-α (#2295s) were purchased from Cell Signaling (Danvers, MA, USA). Isobutylmethylxanthine (IBMX, l7018), dexamethasone (D4902), and Oil red O (O0625) were purchased from Sigma-Aldrich (St. Louis, MO, USA). Orz (152-01272) was purchased from Wako Pure Chemical Industries (Osaka, Japan).

### 2.2. Cell Culture

3T3-L1 mouse fibroblast cells (American Type Culture Collection Manassas, VA, USA) were cultured in DMEM with 10% CS and 1% penicillin-streptomycin-l-glutamine at 37 °C in 5% CO_2_. C2C12 mouse adherent myoblasts cells were cultured in DMEM with 10% FBS and 1% penicillin-streptomycin at 37 °C in 5% CO_2_.

### 2.3. Cell Viability Assay

The 3T3-L1 preadipocytes were seeded in a 96-well plate at a density of 4 × 10^3^ cells/well. After the cells were preconditioning for 24 h, they incubated with various concentrations of Orz for 24 or 48 h. Subsequently, 10 µL of 3-(4,5-dimethylthiazol-2-yl)-2,5-diphenyltetrazolium bromide (MTT) solution in phosphate-buffered saline (PBS) was added to each well and then further incubated at 37 °C for 3 h to detect cell survival. Cell viability was determined by measuring the absorbance using a microplate reader (Tecan, Infinite M200, Männedorf, Switzerland) at 450 nm.

### 2.4. Cell Differentiation of 3T3-L1 Preadipocytes and Oil Red O Staining

The 3T3-L1 preadipocytes were seeded in a six-well plate at a density of 4 × 10^5^ cells/well. Two days after the cells had attained confluence, they were treated with various concentrations of Orz and medium containing 0.5 mM of 3-IBMX, 1 µM of dexamethasone and 1 µg/mL of insulin (MDI) in DMEM with 10% FBS for 2 days. The medium was then changed to DMEM containing 10% FBS, 1 µg/mL of insulin, and Orz for 2 days, and this was changed to fresh DMEM with 10% FBS every 2 days. Eight days after differentiation was induced, the cells were fixed with 10% formaldehyde and stained with a solution of 0.5% Oil Red O in 60% isopropanol. The stained cells were washed with distilled water and observed under a fluorescence microscope. The Oil Red O was extracted from cells with isopropanol and the absorbance was measured at 490 nm.

### 2.5. Glucose Uptake

The 3T3-L1 cells were seeded in a 12-well plate at a density of 2 × 10^5^ cells/well and when they had attained confluence, were treated with Orz for 18 h. The cells were then incubated with 500 μM of palmitic acid (PA) in 2% BSA for 6 h, followed by treatment with 100 μM of insulin in Krebs-Ringer-HEPES for 10 min and 50 μM of 2-(*N*-(7-nitrobenz-2-oxa-1,3-diazol-4-yl)amino)-2-deoxyglucose (2-NBDG) for 15 min. The cellular uptake of 2-NBDG was measured using a fluorometer at excitation and emission wavelengths of 465 and 540 nm, respectively.

### 2.6. Measurement of GLUT4*myc* Translocation in L6 Myotubes

L6 muscle cells stably expressing myc-tagged GLUT4myc (L6-GLUT4myc cells) were kindly provided by Prof. Sungho Ryu, Postech, Pohang-si, South Korea. The cells were maintained in α-minimum essential medium (MEM) supplemented with 10% FBS, 100 units/mL penicillin, and 100 μg/mL streptomycin and grown at 37 °C in 95% humidified air with 5% CO_2_. The cells were pretreated with Orz for 24 h and then starved for 3 h. The positive control cells were treated with 100 nM of insulin for 20 min. The level of myc-tagged GLUT4 at the surface of the intact cells was measured using an antibody-coupled colorimetric assay, which was previously validated [18]. Briefly, following a 10-min incubation with 5% goat serum in PBS, the monolayer of myotubes was exposed to the anti-myc antibody (1:500) for 60 min, fixed with 3% paraformaldehyde for 10 min, and then incubated with horseradish peroxidase (HRP)-conjugated goat anti-mouse IgG (1:1000) for 30 min, at 4 °C. Cells were washed, and incubated with 1 mL of *O*-phenylenediamine reagent for 30 min at room temperature. The reaction was stopped by adding 0.25 mL of 3N hydrochloric acid (HCl). The supernatant was collected, and the absorbance measured at 492 nm. The nonspecific IgG binding was measured using a peroxidase-conjugated anti-mouse IgG and subtracted from the experimental values.

### 2.7. Immunoblotting

The cells were seeded in six-well plates at a density of 2 × 10^5^ cells/well. After 24 h, the cells were starved overnight and then treated with Orz for the indicated times. The cells were lysed with a lysis buffer containing 40 mM HEPES (pH 7.4), 120 mM sodium chloride (NaCl), 1 mM ethylenediaminetetraacetic acid (EDTA), 50 mM sodium fluoride (NaF), 1.5 mM sodium orthovanadate (Na_3_VO_4_), 10 mM β-glycerophosphate, and 1% Triton X-100 supplemented with EDTA-free phosphatase and protease inhibitor cocktail (#78441, Thermo Fisher Scientific Inc., Rockford, IL, USA). After centrifugation at 14,000× *g* for 20 min at 4 °C, the supernatants were boiled in sodium dodecyl sulfate (SDS)-loading buffer. The protein samples were loaded onto Tris-glycine gels, transferred to polyvinylidene fluoride membranes, and probed with the indicated polyclonal or monoclonal antibodies.

### 2.8. Statistical Analysis

The results are presented as mean ± standard deviation (SD). The differences between groups were evaluated using one-way analysis of variance (ANOVA) and Bonferroni *post hoc* test using the GraphPad Prism 5 software (San Diego, CA, USA).

## 3. Results

### 3.1. Orz Enhances Differentiation of 3T3-L1 Preadipocyte

**Figure 1:**
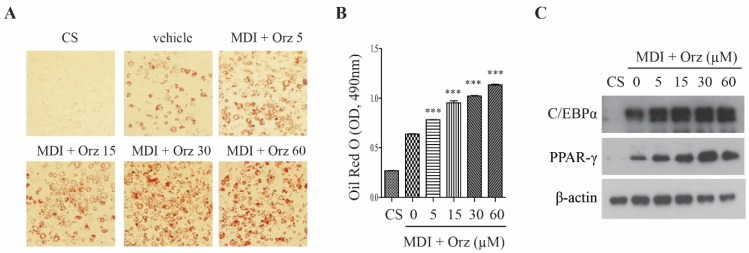
Effects of γ-oryzanol (Orz) on adipocyte differentiation in 3T3-L1 adipocyte cells. 3T3-L1 cells were treated with Orz during adipocyte differentiation for 8 days. (**A**) Lipid accumulation was visualized using Oil Red O staining. (**B**) Quantification of the relative intensity of the Oil Red O, measured at a wavelength of 490 nm. (**C**) Protein expression was analyzed using immunoblot with C/EBPα and PPAR-γ antibodies. Values are means ± SEM, *n* = 3, * *p* < 0.05; **p < 0.01; *** *p* < 0.001 compared to vehicle. PPAR-γ, peroxisome proliferator-activated receptor gamma; C/EBPα, CCAAT/enhanced binding protein alpha. CS, normal medium with calf serum. MDI, medium containing 0.5 mM of 3-IBMX, 1 μM of dexamethasone and 1 µg/mL of insulin.

To determine whether Orz affects differentiation of 3T3-L1 preadipocytes, cells were incubated with MDI media in the presence or absence of Orz. Eight days following the initiation of differentiation, the cells were stained with Oil Red O, which demonstrated that Orz increased lipid accumulation (Figure 1A). This was also supported by quantitative analysis (Figure 1B). To determine whether the increased lipid accumulation induced by Orz was related to adipogenesis, we examined the effects of Orz on C/EBPα and PPAR-γ expression. As expected, Orz increased the C/EBPα and PPAR-γ protein expression (Figure 1C). Our data suggest that Orz enhances cell differentiation by the up-regulation of adipogenesis.

### 3.2. Orz Stimulates Glucose Uptake

**Figure 2:**
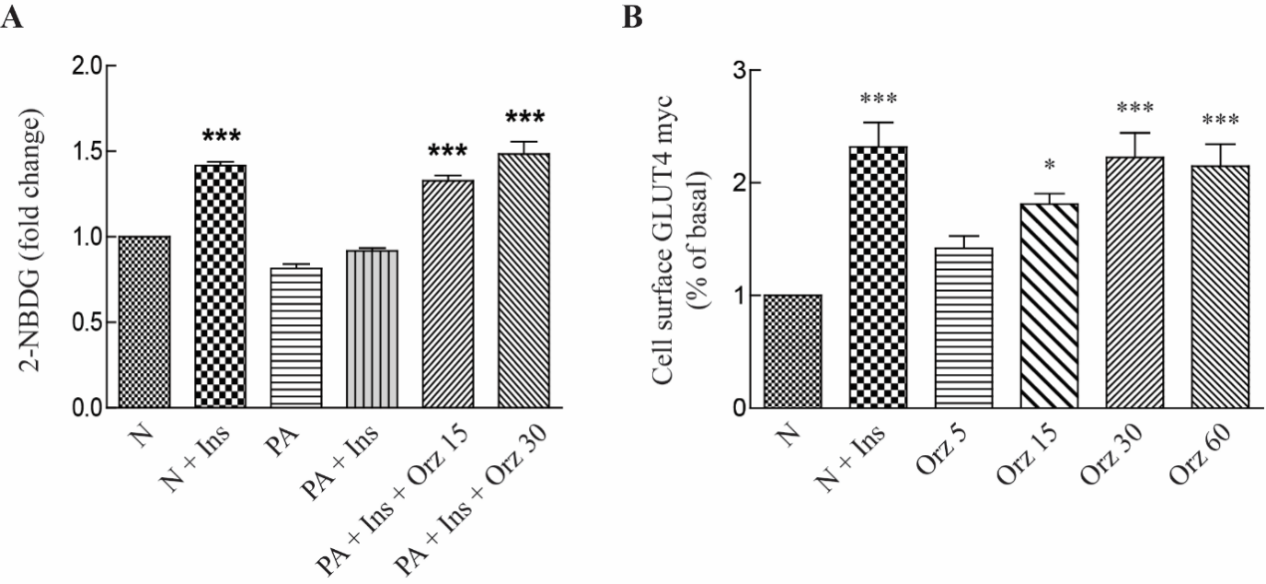
Effects γ-oryzanol (Orz) on glucose uptake and glucose transporter type 4 (GLUT4) translocation. (**A**) Effects of Orz on glucose uptake. 3T3-L1 cells were treated with various concentration of Orz for 18 h. Cells were incubated with 100 nM insulin and 50 μM 2-NBDG for 15 min, followed by incubation with 0.5 mM palmitate for 6 h and then fluorescence intensity of cellular 2-NBDG was measured. (**B**) GLUT4 translocation to the cell membrane was measured in L6-GLUT4myc. Cells were treated with Orz for 24 h or insulin for 10 min. Values are means ± SEM, *n* = 3, * *p* < 0.05; ** *p* < 0.01; *** *p* < 0.001 compared to normal control. 2-NBDG, 2-(*N*-(7-nitrobenz-2-oxa-1,3-diazol-4-yl)amino)-2-deoxyglucose; PA, palmitate; N, normal; Ins, insulin.

Orz is known to increase PPAR-γ protein expression in 3T3-L1 adipocytes, and the activation of PPAR-γ markedly improves insulin sensitivity and glucose tolerance in type 2 diabetes. The regulation of glucose homeostasis by insulin has been extensively investigated and GLUT4 is the key insulin-dependent glucose transporter that responds to insulin binding to the insulin receptor (IR). Palmitic acid causes insulin resistance due to changes in the levels of phosphorylation of the IR and insulin receptor substrate-1 (IRS-1) [19]. Therefore, we examined whether Orz improves palmitate-induced insulin resistance. Exposure to palmitic acid reduced the insulin-stimulated glucose uptake while Orz treatment reversed this effect (Figure 2A). In addition, as expected, treatment with Orz increased GLUT4 translocation in L6-GLUT4myc myoblast dose-dependently (Figure 2B).

### 3.3. Orz-Induced Promotion of Cell Differentiation is mTORC1 Dependent

**Figure 3:**
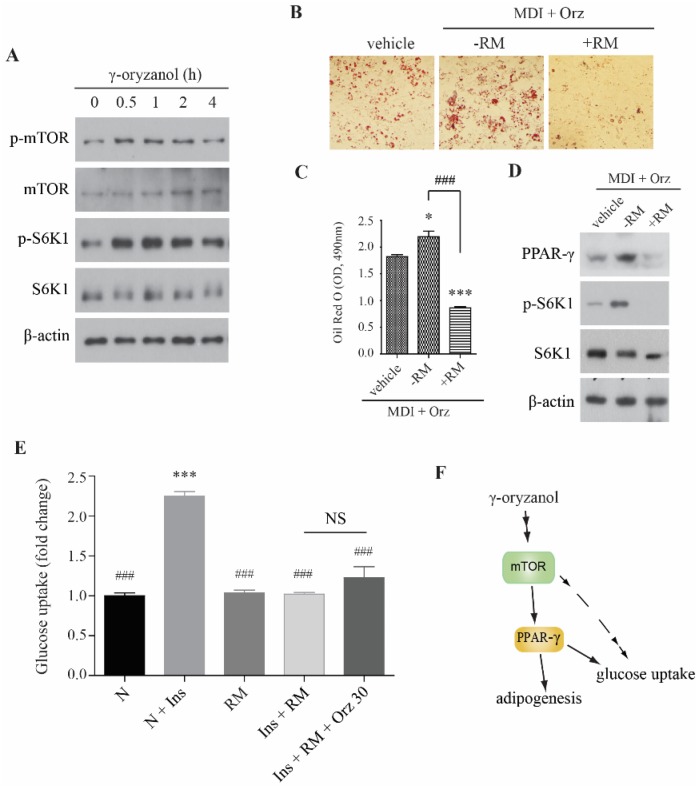
Effects of γ-oryzanol (Orz) on mammalian target of rapamycin (mTOR) signaling in 3T3-L1 adipocyte cells. (**A**) Orz enhances mTOR signaling in 3T3-L1 preadipocytes. Cells were treated with 30 μM Orz for indicated times. (**B**) Orz promotes cell differentiation in mTOR-dependent fashion. 3T3-L1 cells were treated with Orz during adipocyte differentiation for 8 days. Rapamycin (RM) was added from day 0 to 2. Lipid accumulation was visualized by Oil Red O staining. (**C**) Quantification of the relative intensity of the Oil Red O, at a wavelength of 490 nm. Values are means ± SEM, *n* = 3, * *p* < 0.05; *** *p* < 0.001 compared to vehicle. ^###^ *p* < 0.001 compared to rapamycin treated cells. (**D**) Protein expression was analyzed using immunoblot with phospho-S6K1, S6K1, PPAR-γ, and β-actin antibodies. S6K1, 70-kDa ribosomal S6 kinase; PPAR-γ, peroxisome proliferator-activated receptor gamma. (**E**) Rapamycin (RM) co-treatment reduces glucose uptake. 3T3-L1 cells were treated Orz for 18 h. Cells were incubated with 100 nM insulin (Ins) and 50 μM 2-NBDG for 15 min, followed by incubation with or without RM for 1 h and then fluorescence intensity of cellular 2-NBDG was measured. (**F**) Proposed mechanism for the effect of Orz on adipogenesis and glucose uptake. Values are means ± SEM, *n* = 3, *** *p* < 0.001 compared to vehicle. ^###^ *p* < 0.001 compared to N + Ins group.

PPAR-γ is the main regulator of adipogenesis. Recent findings suggest that mTOR regulates adipogenesis by controlling PPAR-γ and sterol regulatory element-binding protein-1 (SREBP-1) [20]. Therefore, we examined whether the Orz-induced changes in cell differentiation of 3T3-L1 preadipocytes correlates with mTOR complex 1 (mTORC1). The results showed that Orz dose-dependently increased the phosphorylation of mTORC1 and S6K1 in 3T3-L1 preadipocytes (Figure 3A), suggesting that Orz may stimulate PPAR-γ expression in adipocytes. To determine whether the Orz-induced cell differentiation was mediated by mTORC1, we co-treated cells with rapamycin (RM) during the cell differentiation procedure. Although Orz enhanced adipocytes differentiation more than the vehicle did, rapamycin co-treatment inhibited lipid accumulation (Figure 3B,C). This result suggests that the enhancement of cell differentiation by Orz is dependent on the activity of mTORC1. The immunoblot analysis also indicated that the phosphorylation of S6K1 was increased by Orz treatment; however, rapamycin suppressed the Orz-induced effect by regulating mTORC1 activity (Figure 3D). Rapamycin co-treatment also suppressed glucose uptake, which is not recovered by Orz (Figure 3E), suggesting that glucose uptake by Orz is dependent on mTORC1 activity.

## 4. Discussion

A number of studies have reported that the consumption of brown rice has potential health benefit effects against obesity-associated metabolic disorders including glucose intolerance and insulin resistance [17,21,22]. Orz is one of the phytochemicals found in brown rice, and it plays an important role in the metabolic benefits of brown rice. In this present study, Orz stimulated cell differentiation by stimulating adipogenic genes including PPAR-γ, which is known to be a major regulator of adipocyte differentiation. In addition, PPAR-γ plays an important role in lipid metabolism and glucose homeostasis. Although we did not evaluate the properties of Orz as a PPAR-γ ligand, our result suggests that it may have agonistic effects similar to those of the potent endogenous PPAR-γ ligand. A recent study has shown that brown rice elicits PPAR-γ agonist activity as part of its anti-diabetic effects [23] and, therefore, Orz may stimulate the activity of PPAR-γ.

Insulin-stimulated glucose transport is important for the control of plasma glucose levels, and this process is severely disrupted in type 2 diabetes and other insulin-resistant states [24]. A study previously reported that Orz reduced the risk of high-fat diet-induced hyperglycemia via regulation of insulin secretion as well as the activities of hepatic glucose-regulating enzymes such as G6Pase and phosphoenolpyruvate carboxykinase (PEPCK) [25]. In the present study, we showed that Orz treatment stimulates GLUT4 translocation into the plasma membrane and glucose uptake concentration-dependently, suggesting this might be the mechanism by which Orz regulates hyperglycemia. Glucose transport is dependent on a variety of signaling events. In adipocytes, glucose transport requires insulin receptor-mediated tyrosine phosphorylation of IRS-1 and IRS-2 and subsequent activation of phosphatidylinositol-4,5-bisphosphate (PI) 3-kinase [26]. Brown rice reportedly increased the IR and IRS-1 proteins [27]. Although Orz itself did not affect the phosphorylation of these proteins, it is possible that it regulated them. Glycogen synthase kinase-3 (GSK-3) has been implicated in various aspects of glucose transport regulation. GSK-3 inhibits mTORC1 signaling via phosphorylation of the tuberous sclerosis complex subunit 2 (TSC2) tumor suppressor [28]. Therefore, mTORC1 signaling is an important positive regulator of GLUT expression and glucose uptake.

The mTOR signaling may be vital for proper insulin signaling in patients with diabetes since the loss of mTOR leads to hypoinsulinemia, glucose intolerance, insulin insensitivity to glucose secretion, and a decrease in β-cell size [29,30]. Increased phosphorylation of S6K1 and 4EBP1 in the pancreatic β-cells of mice improved insulin secretion and resistance to streptozotocin toxicity and obesity [31]. Recent findings have indicated that mTORC1 regulates adipogenesis and lipogenesis by inducing the translation of RNA encoding key constituents of the adipogenic process namely PPAR-γ and C/EBP-α [20]. These observations suggest that mTORC1 signaling plays a key role in type 2 diabetes as well as in adipogenesis and lipogenesis. In this study, Orz positively regulated mTORC1 signaling in preadipocytes and increased the phosphorylation of S6K1 during cell differentiation. This result suggests that Orz may activate PPAR-γ by regulating mTORC1 during adipocyte differentiation.

## 5. Conclusions

In conclusion, the present study shows that Orz induces adipocyte differentiation through the induction of PPAR-γ and C/EBPα expression, which may be mediated via the activity of mTORC1 (Figure 3F). Although there is insufficient evidence to show significant effects, Orz may stimulate glucose uptake via regulation of mTORC1 signaling. Collectively, our results suggest that Orz might be an important constituent for the health benefits of brown rice in obesity-associated metabolic disorders.
